# Professional qualifications of medical affairs pharmaceutical physicians and other internal stakeholders in the pharmaceutical industry

**DOI:** 10.12688/f1000research.123182.1

**Published:** 2022-07-22

**Authors:** Ravi Jandhyala

**Affiliations:** 1 Medialis Ltd, London, SE1 9NH, UK; 2 Centre for Pharmaceutical Medicine Research, King's College University, London, UK

**Keywords:** Medical affairs, pharmaceutical physicians, degree qualifications, decision-making

## Abstract

**Background: **Medical affairs pharmaceutical physicians (MAPPs) have unique value to pharmaceutical companies due to their accountability for activities that benefit regulators, payors, prescribers and patients. This study assessed whether MAPPs’ specialist training and education in pharmaceutical medicine could account for this level of value by determining whether there was significant variation in education and training between MAPPs and other internal stakeholders of pharmaceutical companies.

**Methods: **A systematic search of LinkedIn profiles from the 10 pharmaceutical companies by revenue was conducted between June and October 2021. Job title and type and year of undergraduate and postgraduate qualifications were extracted. A one-sided Mann-Whitney test assessed for differences in the total number of qualifications between MAPPs and other internal stakeholders involved in medical affairs using MAPPs as the reference group. Other internal stakeholders included medical affairs pharmacists (MAPharm), other medical affairs professionals (MAOth), and market access (MAcc), commercial (COmm) and sales professionals. Sub-group analysis determined differences in undergraduate and postgraduate education.

**Results: **In total, 524 profiles were included. Compared to all other internal stakeholders, MAPPs had a significantly higher number of undergraduate (p < 0.001) and postgraduate (MAPharm, p = 0.003; MAOth, p = 0.004; MAcc, COmm and Sales, p < 0.001) qualifications. Additionally, MAPPs had a significantly longer time to industry than other internal stakeholders apart from MAPharm. Of those with clinical qualifications, MAPPs were almost twice as likely to have business qualifications.

**Conclusions: **Of all internal stakeholders, MAPPs had the highest number of qualifications and the best match between expertise and the contextual demands of decision-makers in the pharmaceutical industry. Pharmaceutical companies in the UK can use these findings to clarify role boundaries and decision-making power based on the nature and level of expertise of each internal stakeholder.

## Introduction

The pharmaceutical industry is a unique context due to the need to balance both commercial and patient interests. The idea that organisational survival is predicated on commercial success is a well-established business principle within and beyond the pharmaceutical industry; however, the nature and importance of pharmaceutical medicine expertise is less well-recognised, ^1^ which may have problematic implications for interprofessional collaboration ^2^ and patient-centricity. ^3^ The International Federation of Associations of Pharmaceutical Physicians and Pharmaceutical Medicine and other leading experts ^4^^,^^5^ have advocated for the role of medical affairs pharmaceutical physicians (MAPPs) and defined its competencies, activities and importance to drug development, adoption and post-adoption and launch processes. ^6^^,^^7^ MAPPs aim to identify gaps between practice and evidence-based guidelines, ^8^^–^^13^ improve patient outcomes and decrease healthcare costs. ^14^^–^^16^ Additionally, MAPPs optimise medicine adoption by facilitating engagements between pharmaceutical companies and regulators, payors and prescribers ^17^ and may hold greater weight with prescribers than those in non-medically qualified roles. ^18^

Collaboration between stakeholders in the pharmaceutical industry ^17^^,^^19^ is necessary for optimal medicine prescribing and to improve patients’ health, ^20^ so engagement with regulators, payors and prescribers is vital to pharmaceutical company success. The value of medical affairs pharmaceutical physicians (MAPPs) to pharmaceutical companies has been established as distinct from that of other internal stakeholders due to their unique accountability for activities that benefit all external stakeholders (regulators, payors, prescribers, and patients). ^21^ However, the reasons why they are able to make such a unique contribution have not been characterised. Medical affairs pharmaceutical physicians (MAPPs) have education and training in competencies that cover the entire scope of medical affairs practice and medicine adoption, ^21^^–^^25^ while other stakeholders may have more general educational backgrounds supporting competencies that cover a smaller number of specialist domains ( Table 1). ^26^^–^^29^ Therefore, it was hypothesised that variation in educational background may account for MAPPs’ unique value to pharmaceutical companies.

**Table 1.** Role scope and competencies of physician and non-physician specialists in medical affairs.

Title	Qualifications/skills	Competencies
Medical affairs pharmaceutical physician	Medical degree plus post-graduate pharmaceutical medicine specialty training. ^25^ Specialist training in medical affairs is available.	Drug discovery and development, clinical trials, medicines regulation, pharmacovigilance, ethics, healthcare marketplace, communication and management. ^25^
Medical information specialist	Undergraduate degree in medicine, pharmacy or the life sciences; postgraduate degree in medical information or drug information (not essential). ^26^ Communication skills, medical writing skills, and literature evaluation skills are needed. ^27^	Answering medical information questions, collecting adverse events and product complaints, ^27^ reviewing promotional materials. ^26^
Medical science liaison	Advanced degree (often medical, pharmacy or scientific PhD), scientific and technical expertise, business acumen, communication and interpersonal skills are needed. ^28^	Scientific engagement with key opinion leaders, evidence generation, insight gathering. ^29^

The aim of this study was to determine whether significant variation in qualifications existed between MAPPs and internal stakeholders in the pharmaceutical industry, which could explain their unique value to pharmaceutical companies.

## Methods

A systematic search and comparative quantitative analysis of social media data was conducted prospectively according to a predefined protocol. No specific guidelines were consulted. LinkedIn was searched between June and October 2021 for profiles of professionals in the pharmaceutical industry, including MAPPs, medical affairs pharmacists (MAPharms), other medical affairs professionals (MAOths) and market access (MAcc), commercial (COmm) and sales professionals (for search strings, see Table 2). As with all self-report data, consideration was given to the likely correctness and completeness of information provided on LinkedIn profiles. In the absence of data suggesting potential skew, we assumed that self-reported data quality would fall within a normal distribution, with the majority within acceptable limits and comparable to other self-report data. Additionally, a study assessing the criterion validity of LinkedIn profile elements suggested that it can be used as an accurate source of data on professionals’ qualifications. ^30^ LinkedIn has also been reported as more accurate than other forms of biodata, such as resumes, ^31^ possibly due to the group regulation of individual behaviour according to expectations of adherence to social norms, such as honesty. ^32^

**Table 2.** Search strings.

Search string	Number of hits
Medical affairs AND United Kingdom (Location) AND Company	1900
Medical affairs pharmaceutical physician AND United Kingdom (Location) AND Company	135
Medical affairs MSL AND United Kingdom (Location) AND Company	97
Medical affairs Pharmacist AND United Kingdom (Location) AND Company	499
Medical affairs medical science Liaison AND United Kingdom (Location) AND Company	213
Medical affairs Pharmacy AND United Kingdom (Location) AND Company	355
Medical sales representative AND United Kingdom (Location) AND Company	1000
Pharmaceutical sales representative AND United Kingdom (Location) AND Company	1100
Pharmaceutical market access AND United Kingdom (Location) AND Company	1900
Pharmaceutical commercial AND United Kingdom (Location) AND Company	2100

Note: For company, each of the pharmaceutical companies included in the sample were included consecutively in the search string.

To be included, profiles needed to belong to a professional who was employed at one of the 10 largest pharmaceutical companies by revenue at the time of study, in particular Pfizer, GlaxoSmithKline, Merck & Co., Bayer Pharmaceuticals, Roche, Abbvie, Johnson & Johnson, Sanofi, Gilead Sciences and Novartis. It was assumed that these pharmaceutical companies would have sufficient organisational similarity as to ensure a broad likeness of job descriptions, person specifications and educational requirements for employment within the study sample. The sample of profiles was limited to those whose qualifications were recognised by UK employers to ensure a baseline level of comparability. Data on their undergraduate and postgraduate education (type of degree and year of qualification), date of entry into the pharmaceutical industry, relative age, and research experience were extracted by two research analysts and reviewed by an experienced systematic reviewer to provide outcome data, using MS Excel, comprising the number of degrees and years to first employment in the pharmaceutical industry.

### Statistical analysis

The research hypothesis was tested using a one-sided Mann-Whitney test to assess whether the total number of degrees varied significantly between MAPPs and the other internal stakeholders, using MAPPs as the reference group. Sub-group analysis was performed to determine differences in the level of education (undergraduate and postgraduate) of MAPPs versus other internal stakeholders. The analysis was extended to determine the number of years taken by professionals to gain entry into the pharmaceutical industry, which was taken as a measure of how well the industry recognised their expertise. This was calculated by subtracting the year in which a professional graduated from their first degree/qualification from the year in which professionals took their first job at a pharmaceutical company. If obtained values were negative, then they were statistically adjusted by replacing the year at graduation with the year of entry to ensure that all values were above zero. All statistical analyses were performed using R v4.1.1, and p-values < 0.05 were considered statistically significant.

## Results

### Systematic search of LinkedIn profiles

**Figure 1.**
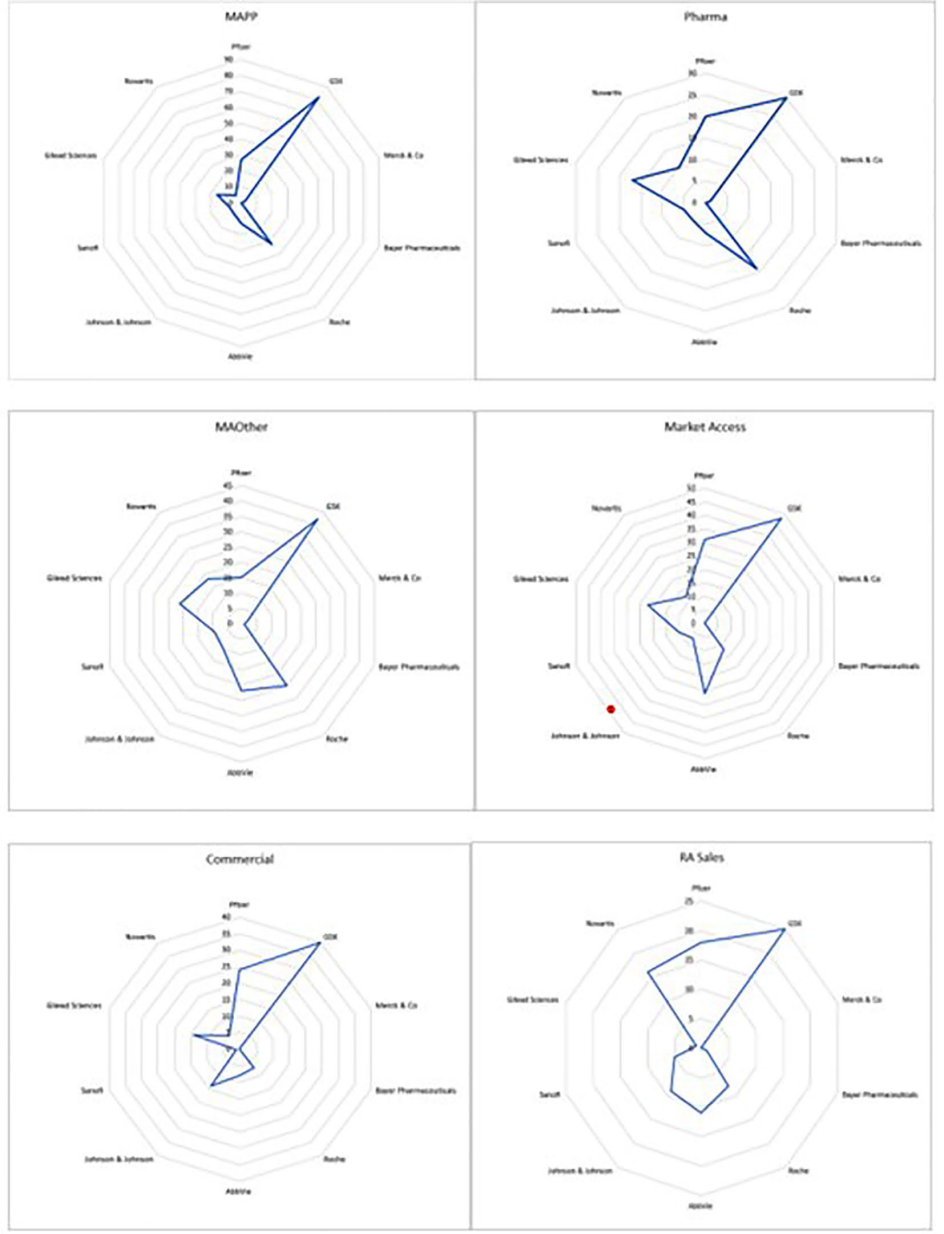
Number of internal stakeholders in each group from the 10 pharmaceutical companies with the highest market share in the UK at the time of study.

The search yielded 9299 profiles with 1209 unique profiles meeting inclusion criteria for review. Of these, 685 were excluded due to missing educational data, unavailability of profiles, professionals not being in primary employment at a pharmaceutical company and professionals being employed in non-medical affairs functions, such as clinical development, regulatory affairs and so on. In total, 524 profiles were included across MAPPs (88), MAPharm (66), MAOth (86), MAcc (97), COmm (94) and sales (93). GlaxoSmithKline was the most represented employer, followed by Abbvie and Roche, with fewest participants being employed by Bayer Pharmaceuticals ( Figure 1).

As shown in Table 3, most qualifications were in the area of science and scientific research (BSc, n = 261; MSc, n = 150; PhD, n = 80), followed by pharmacy (BPharm, n = 40; MPharm, n = 58; PharmD, n = 9), medicine (MBBS/MBChB/BMBCh, n = 68; MD, n = 34) and business (MBA, n = 60). Business and either science, medical or pharmacy qualifications were found in all study groups, but of those with clinical qualifications, MAPPs were almost twice as likely to have business qualifications in the form of MBAs. Of all groups, professionals in the MAoth group were about twice as likely as MAPPs and MPharms and three times as likely as MAcc professionals to have scientific research expertise. Sales and COmm professionals had the least amount of clinical and research expertise, although the COmm group had one of the highest frequencies of business qualifications.

**Table 3.** Frequency of qualifications among all internal stakeholders.

Study group	Frequency of qualification															
	Undergraduate								Postgraduate							
	BTech	BSc	Ba	BSc Nurse	BPharm	MBBS	BMBCh	MBChB	PgDip	PgCert	MSc	MBA	MPharm	PharmD	MD	PhD
MAPP	0	27	1	0	1	40	3	24	31	8	22	12	0	0	28	14
MAPharm	0	11	0	0	19	0	0	0	18	12	22	7	43	3	0	19
MAOth	0	63	1	1	5	0	0	0	1	4	40	7	7	0	2	36
MAcc	0	57	9	2	12	0	0	1	11	6	39	17	6	3	3	9
COmm	0	47	16	0	3	0	0	0	6	3	30	16	0	3	0	2
Sales	2	60	6	1	0	0	0	0	2	6	8	1	2	0	1	0
Total	2	261	33	4	40	40	3	25	63	36	150	60	58	9	34	80

### Difference in number of qualifications and years to industry after first degree between MAPPs and other internal stakeholders

**Figure 2.**
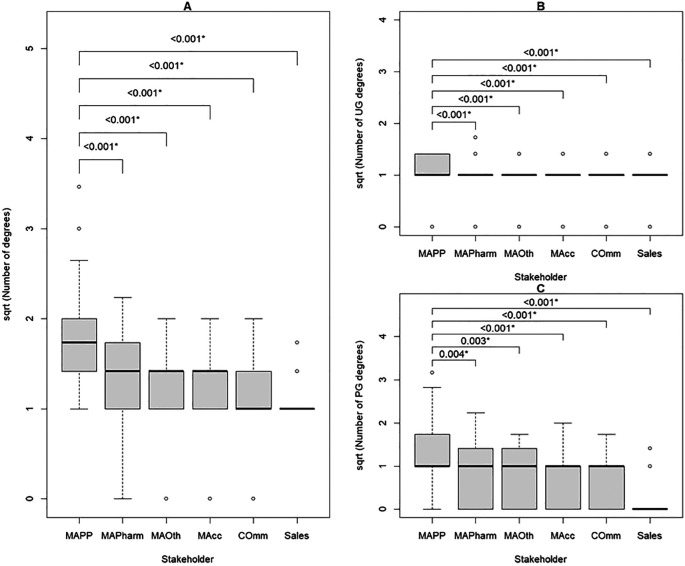
Number of degrees held by internal stakeholders in the pharmaceutical industry; number of degrees transformed using square root to overcome right skewness.

**Figure 3.**
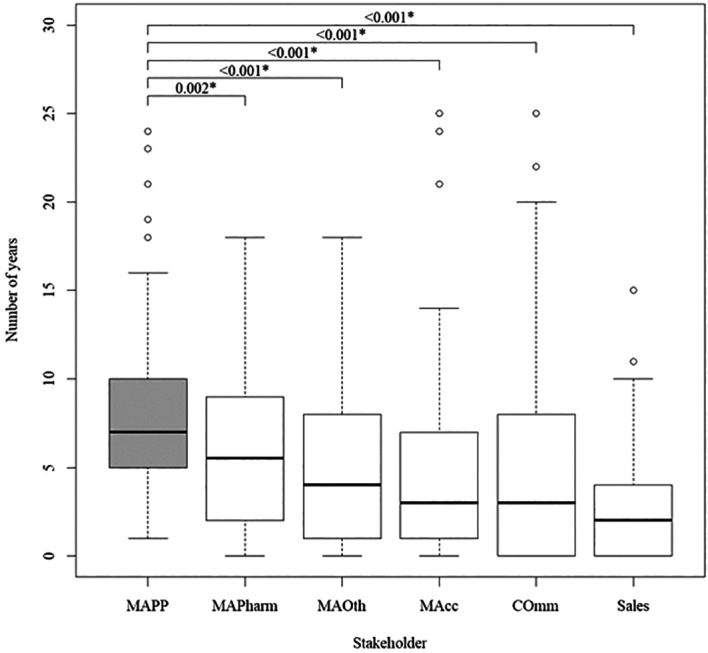
Number of years after completion of first degree for internal stakeholders to gain entry into the pharmaceutical industry, with MAPPs as the reference group.

Of all internal stakeholders, MAPPs had the highest median number of degree qualifications, followed by MAPharms, MAOth and MAcc. Commercial and sales had the lowest. MAPPs had a significantly higher number of total degrees than all other stakeholders ( Table 4, Figure 2a). Subgroup analysis showed that MAPPs had significantly more undergraduate and postgraduate degrees than all other internal stakeholders, but the difference between MAPPs and MAPharm and MAOth was at a lower significance level ( Table 4, Figure 2b). It took MAPPs significantly longer after their first degree to enter the pharmaceutical industry than all other internal stakeholders, but the difference from MAPharm was at a lower significance level ( Figure 3).

**Table 4.** Difference in number of degree qualifications between MAPPs and other internal stakeholders.

Stakeholder	Median	All/total	Undergraduate	Postgraduate
MAPP	3	-	-	-
MA Pharm	2	<0.001 *	<0.001 *	0.004 **
MA Other	2	<0.001 *	<0.001 *	0.003 **
MAcc	2	<0.001 *	<0.001 *	<0.001 *
COmm	1	<0.001 *	<0.001 *	<0.001 *
Sales	1	<0.001 *	<0.001 *	<0.001 *

* p < 0.001.

** p ≤ 0.005.

## Discussion

Historical tensions between commercial and scientific interests in the pharmaceutical industry have created interprofessional working conditions that may have a negative impact on patient and organisational outcomes. Matching human capital with contextual demands for expertise and ensuring that decision-making is carried out by appropriately qualified employees may improve outcomes in domains where both clinical and business factors must be taken into account, such as the pharmaceutical industry. To achieve this, it is first necessary to understand the human capital contributed by each internal stakeholder employed by pharmaceutical companies. This study assessed the expertise of internal stakeholders in the pharmaceutical industry and showed that while MAPPs had significantly more total, undergraduate and postgraduate degree qualifications than all other internal stakeholders, non-MAPP stakeholder groups tended to be characterised by specific types of expertise. Of those with clinical expertise, MAPPs were twice as likely to have business expertise in the form of MBAs, making their training most likely to result in competencies that cover all domains necessary for pharmaceutical company success.

MAPPs had significantly more total, undergraduate and postgraduate degree qualifications than all other internal stakeholders although the latter was at a higher significance level for MAcc, COmm and Sales professionals than for MAPharm and MAOth. There is little previous research comparing the number of qualifications of those in the pharmaceutical industry. However, the results were largely in line with previous findings suggesting that of those with an education in healthcare, physicians were by far most likely to seek out postgraduate study. ^33^ The proportion of physicians with management training in this study (13.6%) also reflected earlier findings. ^34^ The qualifications of physicians have not often been compared to those in non-clinical roles, except in the context of hospital leadership. About twice as many professionals in this study had undergraduate degrees and twice as many had master’s or PhD degrees as those in a previous study in a hospital context. ^35^ This may reflect the comparatively greater need for specialist knowledge in the pharmaceutical industry.

Additionally, MAPPs took significantly longer than all other internal stakeholders apart from MAPharm to enter the pharmaceutical industry after the completion of their first degree. The obvious explanation for this is that MAPPs took longer because they spent more years in education and training. For this to be true, it would be expected that the pattern of differences in qualifications of internal stakeholders compared to MAPPs would reflect the pattern of differences in time to industry. However, all but MAOth and MAPharm had significantly fewer degree qualifications than MAPPs at a significance level of p < 0.001, whereas all but MAPharm had significantly less time to industry than MAPPs at a significance level of p < 0.001. The variation could be explained by the higher number of postgraduate diplomas and certificates completed by MAPPs and MAPharms, assuming they were full time courses. As specialist training in pharmaceutical medicine is in the format of postgraduate diplomas and certificates, the data suggests that while MAOth may accrue a higher number of years of experience in the pharmaceutical industry earlier in their careers, MAPPs may be spending this time developing domain-specific expertise applicable to the pharmaceutical industry in specialist training.

A certain number of years of experience is a common entry requirement for job roles in pharmaceutical companies, especially those in leadership, which reflects the assumption that time spent working within a specific industry allows for the development of domain-specific skills and knowledge. This is in line with human capital theory, which suggests that number of years of experience is a measure of expertise. ^36^ However, evidence suggests that it is not just the number of years of experience that matters but what those years are spent doing. For example, job tenure alone was unrelated to four domains of work performance ^36^ and clinical skills, ^37^^,^^38^ with talent alone ^37^ and training alone ^38^ explaining variation in expertise in clinical contexts. Clinical expertise only seems to be related to years of experience when there is access to the necessary learning experiences. ^39^ Thus, there is a need to assess what constitutes valuable learning experiences within medical affairs practice to understand how expertise can best be developed through both experience and training. Additionally, the pharmaceutical industry may consider developing postgraduate on-the-job training programs to attract MAPPs and facilitate the match between training, expertise and role demands.

Despite differences in time to industry, the difference in postgraduate degree qualifications between MAPPs and MPharm/MOth was at a lower significance level than that of other internal stakeholders, which suggested that MAPharm and MOth also held meaningful specialist expertise. In this study, MOth professionals were about twice as likely to have research expertise than MAPPs and MAPharms. This is to be expected, as entry requirements for medical leads, medical science liaisons and medical information specialists often include a PhD qualification. Of the three groups, MAPPs were about twice as likely to have business expertise at postgraduate level. Therefore, they were the most likely to have expertise that covers the domains necessary for pharmaceutical company success, followed by MAPharms. Additionally, physicians trained in both medicine and management may be better leaders, ^34^^,^^40^ which supports the value of this type of dual expertise to the pharmaceutical industry.

The study did not collect data on the subject of internal stakeholders’ PhD qualifications, but it may be interesting to do so in further research to determine the value of these qualifications to role competencies as well as organisational and patient outcomes given the need for a scientific-medical approach in the pharmaceutical industry. ^41^ While COmm and sales had fewest qualifications over the least number of domains, the skills most important for success for these groups may be measured differently, for example, as psychosocial skills, ^42^ rather than as academic and clinical certifications. This may reflect the different nature of their expertise and role competencies. Additionally, training for some roles, such as medical information specialists and medical science liaisons may be appropriately matched to their specific role scope. In any case, it may be helpful to clarify and define role boundaries and organisational decision-making power within pharmaceutical companies to reflect the specific types of expertise of each internal stakeholder.

It is estimated that about 50% of the study population were likely to have profiles on Linkedin, ^43^^,^^44^ so the sample could not be said to be fully representative. Despite this, our findings are sufficient to fulfil the aim of the study to evidence MAPP expertise in the context of expertise in the pharmaceutical industry. Further research using a wider range of recruitment strategies that examines in more detail the comparative qualitative aspects of pharmaceutical professionals’ education may be useful, especially with regards to scope and match to medical affairs competencies and impact on patient, research and organisational outcomes. Although many profiles, especially those of professionals in older age groups, were excluded on the basis of missing data, this limitation is likely to have affected all professional groups similarly, mitigating its impact on findings. However, there has been a general trend towards younger professionals having a greater number of degrees in recent years, ^45^ so the total number of degrees in all groups may be overestimated given the potential skew towards lower ages in this sample. While self-reported education and experience on Linkedin is generally more accurate than other forms of self-report in this context, ^46^ it is possible that profiles were not up-to-date, and the attainment of a degree qualification does not necessarily denote quality scholarship or the amount of time spent studying.

## Conclusions

Of all internal stakeholders, MAPPs had the highest number of qualifications and the longest time to industry as well as the best match between expertise and the contextual demands of decision-makers in the pharmaceutical industry. Pharmaceutical companies can use these findings to clarify role boundaries and decision-making power based on the nature and level of expertise of each internal stakeholder. These findings have provided some explanation for MAPPs’ unique value by suggesting that their educational background is a significant factor distinguishing them from other internal stakeholders. Further study is needed to understand how this education and training may develop expertise that accounts for their unique value, that is, how competencies developed during education and training may be relevant to their work with all external stakeholders. Understanding what constitutes useful learning experiences for developing expertise in medical affairs may be beneficial and could facilitate the development of specialised on-the-job training programs to ensure a match between training, expertise and role demands.

## Ethical approval

This study was exempt from requiring ethical approval under the King’s College of London research ethics guidelines, as no human participants were recruited and unidentifiable human data were collected for this study.

## Data availability

### Underlying data

The underlying data to this research cannot be shared due to the ethical and copyright restrictions surrounding social media data. The Methods section contains detailed information to allow replication of the study. Any queries about the methodology should be directed to the corresponding author.

## Author contributions

RJ conducted the study and developed and approved the manuscript. The author affirms that the manuscript is an honest, accurate, and transparent account of the study being reported; that no important aspects of the study have been omitted, and any discrepancies from the study as planned (and, if relevant, registered) have been explained.
