# Supplementary material for: Professional qualifications of medical affairs pharmaceutical physicians and other internal stakeholders in the pharmaceutical industry
Source: F1000Res. 2022 Nov 7;11:813. Originally published 2022 Jul 22. [Version 2] doi: 10.12688/f1000research.123182.2 (PMC9647148; doi:10.12688/f1000research.123182.2)
Supplement: Peer review history

# Peer review: Reviewer response for version 2

**DOI:** 10.5256/f1000research.140405.r155174

**Published:** 2022-11-09

**Authors:** Jonathan Day

**Affiliations:** 1 BioMarin Pharmaceutical Inc, London, UK

Many thanks to the author for responding to and addressing the comments raised during my primary review. I have no further suggestions and consider my original comments resolved.

Is the work clearly and accurately presented and does it cite the current literature?

Yes

If applicable, is the statistical analysis and its interpretation appropriate?

Yes

Are all the source data underlying the results available to ensure full reproducibility?

No

Is the study design appropriate and is the work technically sound?

Yes

Are the conclusions drawn adequately supported by the results?

Yes

Are sufficient details of methods and analysis provided to allow replication by others?

Yes

Reviewer Expertise:

Clinical Development Physician working in Rare Disease Drug development (Skeletal Dysplasia, Gene Therapy, Bleeding Disorders, Thrombosis and Inflammation). Original background in cardiovascular medicine.

I confirm that I have read this submission and believe that I have an appropriate level of expertise to confirm that it is of an acceptable scientific standard.

# Peer review: Reviewer response for version 2

**DOI:** 10.5256/f1000research.140405.r155175

**Published:** 2022-11-08

**Authors:** Domenico Criscuolo

**Affiliations:** 1 Genovax, Turin, Italy

I have read the amended version, and approved it for indexing.

Is the work clearly and accurately presented and does it cite the current literature?

Yes

If applicable, is the statistical analysis and its interpretation appropriate?

Yes

Are all the source data underlying the results available to ensure full reproducibility?

Yes

Is the study design appropriate and is the work technically sound?

Yes

Are the conclusions drawn adequately supported by the results?

Yes

Are sufficient details of methods and analysis provided to allow replication by others?

Yes

Reviewer Expertise:

I have 47 years’ experience in drug development, with a special focus on Medical Affairs

I confirm that I have read this submission and believe that I have an appropriate level of expertise to confirm that it is of an acceptable scientific standard.

# Peer review: Reviewer response for version 1

**DOI:** 10.5256/f1000research.135265.r145143

**Published:** 2022-10-26

**Authors:** Domenico Criscuolo

**Affiliations:** 1 Genovax, Turin, Italy

Very interesting paper, underlining the importance of high level qualifications for physicians working in the Medical Affairs department of the pharmaceutical industry.

 The author made a careful search via LinkedIn of profiles of professionals in the pharmaceutical industry, and finally selected 524 profiles with full educational details.

 The analysis of these data demonstrate that physicians are the professional group with higher postgraduate education, and this finding is relevant for the role Medical Affairs physicians must cover.

 The manuscript is well written, has a few tables and several references and does not need any revision.

Is the work clearly and accurately presented and does it cite the current literature?

Yes

If applicable, is the statistical analysis and its interpretation appropriate?

Yes

Are all the source data underlying the results available to ensure full reproducibility?

Yes

Is the study design appropriate and is the work technically sound?

Yes

Are the conclusions drawn adequately supported by the results?

Yes

Are sufficient details of methods and analysis provided to allow replication by others?

Yes

Reviewer Expertise:

I have 47 years’ experience in drug development, with a special focus on Medical Affairs

I confirm that I have read this submission and believe that I have an appropriate level of expertise to confirm that it is of an acceptable scientific standard.

# Response

**Published:** 2022-10-26

**Authors:** Ravi Jandhyala

**Affiliations:** Medialis Ltd, UK

Dear Domenico Criscuolo,

 Thank you for the outstanding and positive review of this manuscript. 

 Best wishes,

 Ravi

# Peer review: Reviewer response for version 1

**DOI:** 10.5256/f1000research.135265.r148656

**Published:** 2022-09-12

**Authors:** Jonathan Day

**Affiliations:** 1 BioMarin Pharmaceutical Inc, London, UK

The author analyzed professional qualifications (using a well-known professional social media platform) across different types of employees (Medical Affairs Physicians, Market Access, Commercial, Sales) at pharmaceutical companies across the UK and reports on the significant variation in the number and type of qualifications as well as time taken in education prior to joining the pharmaceutical industry. Those working in Medical Affairs positions tended to have spent more time in education and therefore had a larger number of undergraduate and postgraduate qualifications, including business qualifications (MBA). The author concludes that those with greater levels of education, more qualifications and more relevant experience might be better placed to make decisions that impact clinical as well as business factors across the pharmaceutical industry.    

 Suggestions:

 Figure 1 is not very high resolution (at least in the PDF I have access to) and is therefore difficult to interpret easily. A higher resolution Figure or one with larger font size would be helpful.

 The Discussion does address some of the limitations of this study. The link between qualifications and business impact is somewhat subjective and could do with some statements to this effect. For instance, Medical Affairs professionals might spend more time in education prior to joining the Industry but those who join other functions (such as commercial and sales) will arguably develop other highly relevant skills and experience through their work experience. These competencies would not be captured in the current analysis. Different entry level qualifications at the time of starting a job in the Industry will also likely inform on an employee’s seniority level (and thus decision maker influence) within the organization. Additionally, beyond seniority and qualifications many other competencies will inform on an individual’s degree of influence and success as a decision maker. Attainment of these attributes is not a unique challenge in the pharmaceutical industry and something likely relevant to all sectors.  

Is the work clearly and accurately presented and does it cite the current literature?

Yes

If applicable, is the statistical analysis and its interpretation appropriate?

Yes

Are all the source data underlying the results available to ensure full reproducibility?

No

Is the study design appropriate and is the work technically sound?

Yes

Are the conclusions drawn adequately supported by the results?

Yes

Are sufficient details of methods and analysis provided to allow replication by others?

Yes

Reviewer Expertise:

Clinical Development Physician working in Rare Disease Drug development (Skeletal Dysplasia, Gene Therapy, Bleeding Disorders, Thrombosis and Inflammation). Original background in cardiovascular medicine.

I confirm that I have read this submission and believe that I have an appropriate level of expertise to confirm that it is of an acceptable scientific standard, however I have significant reservations, as outlined above.

# Response

**Published:** 2022-10-27

**Authors:** Ravi Jandhyala

**Affiliations:** Medialis Ltd, UK

Firstly, we would like to take this time to thank the reviewer for their efforts and positive review. A detailed response to the reviewer comments is provided below.

**Comment**: Figure 1 is not very high resolution (at least in the PDF I have access to) and is therefore difficult to interpret easily. A higher resolution Figure or one with larger font size would be helpful.

**Answer: **Thank you for bringing this to our attention. We have revised the image and submitted a higher resolution and more detailed copy alongside this revised manuscript.

**Comment: **Medical Affairs professionals might spend more time in education prior to joining the Industry but those who join other functions (such as commercial and sales) will arguably develop other highly relevant skills and experience through their work experience. These competencies would not be captured in the current analysis. Different entry level qualifications at the time of starting a job in the Industry will also likely inform on an employee’s seniority level (and thus decision maker influence) within the organization. Additionally, beyond seniority and qualifications many other competencies will inform on an individual’s degree of influence and success as a decision maker. Attainment of these attributes is not a unique challenge in the pharmaceutical industry and something likely relevant to all sectors.   

**Answer:** Thank you for the excellent suggestion. We have revised the discussion to address the concerns raised and have included further insights into the qualification and experience attained by MAPPs.****
